# Multicellular “hotspots” harbor high-grade potential in lower-grade gliomas

**DOI:** 10.1093/noajnl/vdab026

**Published:** 2021-02-08

**Authors:** Alastair J Kirby, José P Lavrador, Istvan Bodi, Francesco Vergani, Ranjeev Bhangoo, Keyoumars Ashkan, Gerald T Finnerty

**Affiliations:** 1 Department of Basic and Clinical Neuroscience, King’s College London, London, UK; 2 Department of Neurosurgery, King’s College Hospital NHS Foundation Trust, London, UK; 3 Department of Clinical Neuropathology, King’s College Hospital NHS Foundation Trust, London, UK; 4 Department of Neurology, King’s College Hospital NHS Foundation Trust, London, UK

**Keywords:** brain tumor, glia, malignant progression, nestin, vessel co-option

## Abstract

**Background:**

Lower-grade gliomas may be indolent for many years before developing malignant behavior. The mechanisms underlying malignant progression remain unclear.

**Methods:**

We collected blocks of live human brain tissue donated by people undergoing glioma resection. The tissue blocks extended through the peritumoral cortex and into the glioma. The living human brain tissue was cut into ex vivo brain slices and bathed in 5-aminolevulinic acid (5-ALA). High-grade glioma cells avidly take up 5-ALA and accumulate high levels of the fluorescent metabolite, Protoporphyrin IX (PpIX). We exploited the PpIX fluorescence emitted by higher-grade glioma cells to investigate the earliest stages of malignant progression in lower-grade gliomas.

**Results:**

We found sparsely distributed “hot-spots” of PpIX-positive cells in living lower-grade glioma tissue. Glioma cells and endothelial cells formed part of the PpIX hotspots. Glioma cells in PpIX hotspots were IDH1 mutant and expressed nestin suggesting they had acquired stem-like properties. Spatial analysis with 5-ALA-conjugated quantum dots indicated that these glioma cells replicated adjacent to blood vessels. PpIX hotspots were formed in the absence of angiogenesis.

**Conclusion:**

Our data show that PpIX hotspots represent microdomains of cells with high-grade potential within lower-grade gliomas and identify locations where malignant progression could start.

**Key Points**

- Malignant progression of lower-grade gliomas studied in slices of living human brain tissue.
- Lower-grade gliomas contain hotspots of nestin-positive glioma cells and endothelial cells.
- Glioma hotspots may signify the earliest stages of malignant progression.

**Importance of the Study**

Lower-grade gliomas may be indolent for many years before developing malignant behavior. The mechanisms underlying malignant progression remain unclear. We studied where malignant progression starts in gliomas by using living human brain tissue donated by people undergoing glioma resection. High-grade glioma cells were labeled fluorescently and imaged in living ex vivo human brain slices. We found sparsely distributed groups of glioma cells with high-grade features clustered around small blood vessels, which we termed hotspots. The hotspots occur in the absence of angiogenesis. We propose that the hotspots are the seedbeds for malignant progression.

Gliomas are the commonest primary brain tumor.^1^ A subset of diffuse gliomas are indolent for many years before they adopt more aggressive behavior.^2^ Management of these lower-grade diffuse gliomas is a significant clinical problem because they usually affect young adults. The median survival varies between 5.2 and 7.2 years with only 20% of patients surviving for 2 decades.^3^ Due to the young age at presentation, lower-grade gliomas cause a large loss of “potential years of life.” ^4^

A greater understanding of the mechanisms underlying the malignant progression of lower-grade diffuse gliomas is key to developing new treatments. Previous work has focused on molecular mechanisms, such as new mutations,^5,6^ changes in gene expression,^7,8^ and altered signaling pathways.^9^ However, these studies do not identify the earliest stages of malignant progression. The issue is that they compare a time before malignant progression with a time when malignant progression is established. The studies do not tell us how the transition between the 2 tumor states occurs. Accordingly, we do not know which molecular changes initiate malignant progression, which act to sustain it, and which are the result of malignant progression.

The site where malignant progression begins could give important information about the underlying mechanisms. Identifying the spatial origins of malignant progression would open opportunities to study how it is initiated and the factors that sustain it. However, currently, it is not known whether malignant progression can start in any part of a lower-grade diffuse glioma or whether it occurs at specific locations where the glioma microenvironment fosters malignant progression.

We investigated the spatial origins of malignant progression at a cellular level by using ex vivo human brain tissue. In common with other tumors, high-grade glioma (HGG) cells exhibit metabolic reprogramming.^10^ Glioma cells avidly take up 5-aminolevulinic acid (5-ALA), which is upstream in the heme biosynthesis pathway, and metabolize it to the fluorescent molecule, Protoporphyrin IX (PpIX).^11^ HGG cells accumulate high levels of PpIX, which makes them fluorescent.^11^ Neurosurgeons have taken advantage of 5-ALA-induced PpIX fluorescence to aid resection of higher-grade gliomas intraoperatively.^12^

We exploited the PpIX fluorescence emitted by higher-grade glioma cells to investigate the earliest stages of malignant progression in lower-grade gliomas. We collected blocks of live human brain tissue donated by people undergoing glioma resection and prepared ex vivo brain slices, which preserved the architecture of the glioma and peritumoral cortex. The human brain slices were bathed in 5-ALA to enable us to image cells in the tissue with PpIX fluorescence. We identified sparsely distributed clusters of PpIX fluorescent cells, which we refer to as PpIX hotspots. The PpIX hotspots in lower-grade glioma tissue contained nestin-positive (nestin^+^) glioma cells and endothelial cells. Our data suggest that the PpIX hotspots represent microdomains of cells with high-grade potential within lower-grade gliomas.

## Materials and Methods

### Ethical Approval and Consent

The UK Human Research Authority (https://www.hra.nhs.uk/) approved the collection of ex vivo brain samples following a favorable opinion from the South West Research Ethics Committee (REC approval code: 18/SW/0022).

The ex vivo brain samples were donated by participants who underwent a craniotomy for either tumor resection or intractable epilepsy at King’s College Hospital between March 2018 and December 2019 (Table 1). All participants gave informed consent prior to their surgery. A person was eligible for the study if (s)he required brain surgery that necessarily involved removal of brain tissue to treat the participant’s disease. Individuals who had multiple brain diseases or previous chemotherapy/radiotherapy were excluded. Malignant progression of diffuse lower-grade gliomas was identified preoperatively by neuroimaging, for example, contrast enhancement, increased perfusion, and then confirmed by finding focal, high-grade features neuropathologically. No participants were prescribed anticonvulsant medications, which reduce 5-ALA synthesis in glioma cells.^13^

**Table 1.** Tumour Information and Intraoperative PpIX Fluorescence for Each Participant

ID	Age	Sex	Tumor Information				Operation PpIX Fluorescence
			Site	Diagnostic Grade	Ex vivo Grade	Molecular Markers	
1	65	M	Right frontal	Oligo III	II	IDH1^mut^, 1p/19q co-del, ATRX retained	Negative
2	29	F	Right frontal	Oligo II	II	IDH1^mut^, 1p/19q co-del, ATRX retained, TERTp^mut^	Negative
3	39	F	Left frontal	Oligo III	III	IDH1^mut^, 1p/19q co-del, ATRX retained	Negative
4	31	F	Left frontal	Oligo III	III	IDH1^mut^, 1p/19q co-del, ATRX retained	Negative
5	34	M	Left frontal	Astro III	II	IDH2^mut^ (R172C), ATRX lost, TERTp^wt^	Negative
6	47	M	Right frontal	Oligo III	II	IDH1^mut^, 1p/19q co-del, ATRX retained, TERTp^mut^	Positive
7	27	F	Left frontal	Oligo II	II	IDH1^mut^, 1p/19q co-del, ATRX retained, TERTp^mut^	Negative
8	43	F	Right temporal	Oligo II	II	IDH1^mut^, 1p/19q co-del, ATRX retained	Negative
9	55	F	Right temporal	Astro III	III	IDH1^mut^, ATRX lost, TERTp^wt^	Negative
10	55	M	Right frontal	Oligo III	II	IDH1^mut^, 1p/19q co-del, ATRX retained	Negative
11	52	F	Left parietal	GBM IV	IV	IDH1^wt^, ATRX retained	Positive
12	69	M	Right occipital	GBM IV	IV	IDH1^wt^, ATRX retained, TERTp^mut^	Positive
13	57	M	Right frontal	GBM IV	NGC	IDH1^wt^, ATRX retained, TERTp^mut^	Positive
14	54	F	Right frontal	GBM IV	NGC	IDH1^wt^, TERTp^mut^	Positive
15	62	M	Right temporal	GBM IV	IV	IDH1^wt^, ATRX retained, TERTp^mut^	Positive
16	27	F	Left frontal	GBM IV	IV	IDH1^wt^, ATRX retained, TERTp^mut^	Positive
17	21	M	Right frontal	Focal cortical dysplasia	NGC	CD34 Negative	Negative
18	28	M	Right frontal	Astro III	III	IDH1^mut^, ATRX lost	Positive
19	58	M	Left parietal	Metastatic adenocarcinoma	NGC	N/A	Negative
20	69	F	Left temporal	Astro III	III	IDH1^wt^, ATRX retained, TERTp^wt^	Negative

Oligo, oligodendroglioma; Astro, astrocytoma; GBM, glioblastoma; IDH1^wt^, no R132H mutation in isocitrate dehydrogenase 1; IDH1^mut^, R132H mutation in isocitrate dehydrogenase 1 unless stated; 1p/19q co-del, co-deletion affecting chromosomes 1p and 19q; ATRX, ATP-dependent helicase ATRX; TERTp, telomerase reverse transcriptase promoter.

### Live Human Brain Tissue Collection

Participants with suspected WHO grade III or grade IV gliomas were given 5-ALA (Gliolan, 20 mg/kg) 2–4 h prior to their surgery.

**Figure 1.**
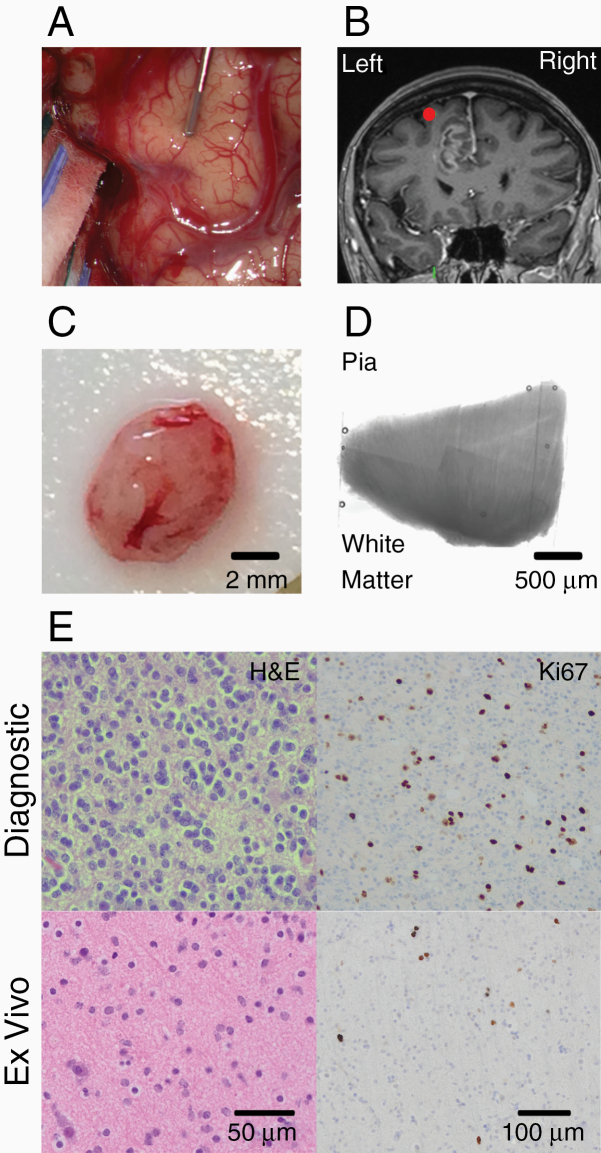
Ex vivo human brain tissue. (A) Intraoperative image of exposed brain surface of a participant with a glioblastoma. A neuronavigation probe lies on the left superior frontal gyrus where the sample was taken from. (B) Intraoperative coronal MRI snapshot taken with the neuronavigation system. The red dot denotes the location of the probe shown in panel A superior to the tumor. (C) Block of brain tissue resected from a participant in A and B. (D) Montage of brightfield images (5×) of a neocortical brain slice cut from block shown in C. Slice spans white matter to pia. (E) H&E and Ki-67 staining of the diagnostic biopsy (top row) and ex vivo brain sample (bottom row).

The location of the brain sample was recorded with the intraoperative neuronavigation system (StealthViz; Medtronic) and a brightfield image of the surface of the brain (Zeiss OPMI Pentero 900 or Zeiss KINEVO 900 operating microscope) before the sample was taken (Figure 1A and B). PpIX fluorescence was detected by switching the operating microscope to fluorescence mode (excitation 400–410 nm, emission 620–710 nm).

Before removing the block of tissue, the surface of the brain was cooled with chilled Ringer’s solution. The ex vivo tissue blocks were excised with a scalpel and immediately placed in cooled dissection artificial cerebral spinal fluid (aCSF) comprising in mM: 108 choline-Cl, 3 KCl, 26 NaHCO_3_, 1.25 NaHPO_4_, 25 d-glucose, 3 Na pyruvate, 2 CaCl_2_, 1 MgCl_2_, and 0.1 Heparin and was bubbled with 95% O_2_/5% CO_2_.

The ex vivo brain tissue was transferred to the laboratory in a transportation system that kept the samples cool while bathing them in dissection aCSF constantly gassed with 95% O_2_/5% CO_2_.^14^ After arriving in the laboratory, the ex vivo tissue blocks were bathed in cooled dissection aCSF and sliced into 300–500 µm thick sections using a vibratome (Campden Instruments; Figure 1C and D). After every fifth section, a 1 mm thick section was cut and fixed with 4% paraformaldehyde in 1 mM phosphate-buffered saline for 2–4 h at 4°C for neuropathology.

### Ex Vivo Imaging

The ex vivo brain slices were transferred to an incubation chamber (Scientific Systems Design). The aCSF in the incubation chamber comprised (mM): 120 NaCl, 3 KCl, 23 NaHCO_3_, 1.25 NaHPO_4_, 10 d-glucose, 2 CaCl_2_, and 1 MgCl_2_ bubbled with 95% O_2_/5% CO_2_. The aCSF was gradually warmed to 37°C and 1 mM 5-ALA (Sigma: 5451-09-2) was added prior to imaging. The ex vivo samples were imaged on an interface recording chamber (Scientific Systems Design) mounted on an Olympus BX51WI epifluorescence microscope. The ex vivo fluorescence and brightfield images were imaged with a Spot RT sCMOS cooled 5MP camera (RT39M5, Spot Imaging) controlled by Spot Advanced imaging software (Spot Imaging).

### 5-ALA-Conjugated Quantum Dots

We conjugated 5-ALA to fluorescence quantum dots nanocrystals following the manufacturer’s instructions (ThermoFisher Qdot Probes; Supplementary Methods). The 5-ALA-conjugated Qdots were incubated with ex vivo brain tissue in aCSF with 1 mM 5-ALA for 4 h. The slices were imaged on an Olympus BX51WI epifluorescence microscope using a Spot RT sCMOS cooled 5MP camera before fixation in 4% PFA for 20 min.

### Ex Vivo PpIX Image Analysis

PpIX fluorescence was quantified in FIJI (https://imagej.net/Fiji) using a custom-written script (Supplementary Methods, Supplementary Figure 1).

### Neuropathological Processing

Brain tissue slices, 1 mm thick, were fixed in 10% formalin and embedded in paraffin blocks. Immunohistochemistry and neuropathological assessment for brain tumors were performed by the Neuropathology Department, King’s College Hospital. Sections were embedded in paraffin blocks and 4–5 µm sections were used for immunohistochemistry. Neuropathology slides were imaged on an Olympus VS120 Slide scanner and quantified using a custom-written script in FIJI (Supplementary Methods, Supplementary Figure 2).

### WHO Grade of Ex Vivo Human Brain Samples

The glioma diagnosis was based on the histopathological features of the diagnostic biopsy from the tumor core. The ex vivo research samples were graded by a Consultant Neuropathologist (I.B.) who was blind to the PpIX fluorescence data (Table 1).

We identified 2 ex vivo samples from WHO grade IV gliomas that had no or infrequent tumor cells (<10 cells/mm^2^). These samples were combined with tissue from one case of focal cortical dysplasia and one case of metastatic adenocarcinoma and used as controls.

### Immunofluorescence

After live imaging was completed, brain slices were fixed, resectioned (50 µm thick), and immunofluorescence staining was performed using standard techniques (Supplementary Methods, Supplementary Table 1). Resectioned slices were imaged on an inverted confocal microscope (Nikon AR1) using a 20× air objective (NA 0.75) and pinhole size of 1.2 Airy units. Z-stacks with 5 µm steps were acquired through the entire slice. The same gain and laser power were used to image each of the fluorophores. Images were acquired using NIS-elements (Nikon) and analyzed with FIJI software (https://imagej.net/Fiji).

### Statistical Analysis

We could not perform all tests on all samples. Therefore, the data used for each figure panel are listed in Supplementary Figure 2. Statistical analysis and graphing were undertaken in Graph Pad Prism 7. Data were described by their mean ± standard error or median and interquartile range. Statistical tests were two-tailed and had a threshold for type 1 statistical error of *α* < 0.05. Means were compared with *t*-tests or a repeated measures ANOVA if the data fulfilled the assumptions for parametric tests. A Mann–Whitney *U*-test was used when parametric tests were not appropriate. The linear regression function was used to fit lines to data that fulfilled the normality and equal variance conditions. A generalized linear model running under R (R project for Statistical Programming, https://www.r-project.org/) was used to fit a linear relationship (quasipoisson family) between PpIX^+^ cells and glioma grade.

## Results

Neuropathological examination of lower-grade gliomas reveals that malignant progression starts focally within the tumor.^15^ Therefore, we investigated whether our ex vivo brain tissue samples, which included the invasive edge of gliomas, had the same neuropathological features as the diagnostic biopsies (Figure 1E). We found that our ex vivo brain samples were frequently classified as having the neuropathological features of a WHO grade II glioma when the diagnostic biopsy was WHO grade III (ex vivo sample/diagnostic biopsy; 4/9 WHO grade III, Table 1, Supplementary Table 2). We refer to our ex vivo brain samples as WHO II {III} when the ex vivo sample was graded as WHO II and the diagnostic biopsy graded as WHO III. In participants with glioblastoma (WHO grade IV), no glioma cells (NGCs) were present in 2 patients’ research samples collected from the 6 participants with glioblastoma.

We reasoned that we could use brain tissue with WHO grade II neuropathological features which was collected from lower-grade gliomas to study the origins of malignant progression.

### PpIX “Hotspots” at the Edge of Lower-Grade Gliomas

Tumor cells exhibit increasing metabolic changes as they become more malignant.^10^ The altered cellular metabolism is a source of biomarkers for tumor cells. Neurosurgeons use the increase in PpIX fluorescence emitted by glioma cells intraoperatively to improve resection of higher-grade gliomas.^12^ More recently, it has been proposed that macroscopic PpIX fluorescence visible during surgery to resect lower-grade gliomas indicates an area of malignant progression.^16^ Therefore, we explored which cells exhibit PpIX fluorescence in adult lower-grade gliomas. We cut blocks of ex vivo human brain tissue donated by patients undergoing glioma resection into brain slices to enable us to image cellular PpIX fluorescence in living tissue.

**Figure 2.**
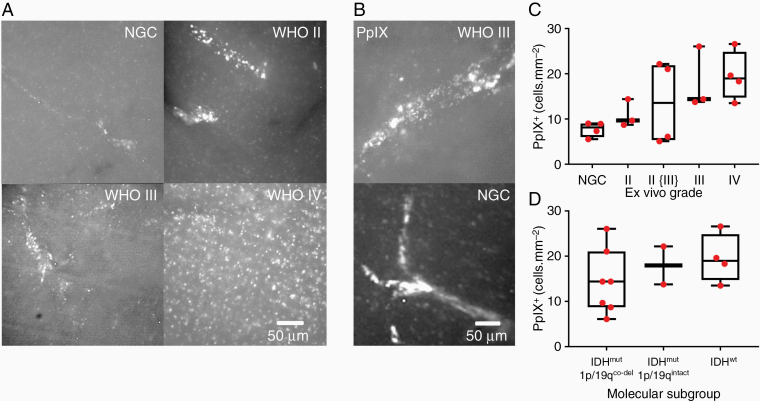
PpIX “hotspots” in lower-grade gliomas. (A) PpIX^+^ cells in *ex vivo* human brain tissue from the control group with no glioma cells (NGC) and from the invasive edge of WHO II–IV gliomas: NGC image, the patient had a glioblastoma. (B) Images (×40) of tubular PpIX structures in WHO grade III glioma (upper) and NGC from a participant with focal cortical dysplasia (lower). (C) PpXI^+^ cell density at glioma edge for different tumor grades. (D) PpIX^+^ cell density for each glioma molecular subgroup.

PpIX is a naturally occurring molecule. Therefore, we first studied PpIX fluorescence in brain tissue that was not infiltrated by glioma cells to establish baseline levels of PpIX fluorescence. We found that PpIX fluorescence in the ex vivo brain tissue that had no glioma cells frequently formed tubular structures resembling blood vessels (Figure 2A; Supplementary Figure 3A and B). We quantified the cells exhibiting PpIX fluorescence using Particle Analysis in FIJI (Supplementary Methods, Supplementary Figure 1). Ex vivo brain samples that had not been infiltrated by glioma contained cells that exhibited PpIX fluorescence (PpIX fluorescent cells = 7.7 ± 0.8 cells/mm^2^, *n* = 4 patients comprising 2 glioblastomas with no glioma cells in the research sample, 1 focal cortical dysplasia, 1 metastatic adenocarcinoma) (Figure 2B and C). Henceforth, any cells exhibiting PpIX fluorescence are termed PpIX^+^.

We then studied ex vivo human brain tissue infiltrated by glioma cells. In lower-grade gliomas, PpIX^+^ cells formed bright cylindrical clusters, which we refer to as “PpIX hotspots” (Figure 2A and B). In contrast, the PpIX^+^ cells at the edge of WHO grade IV gliomas appeared more uniformly spread (Figure 2A; Supplementary Figure 1). We found that the density of PpIX^+^ cells increased with WHO grade (PpIX^+^ cell density, median [IQR]: NGC, 7.7 [6.0–9.0] cells/mm^2^; WHO II, 10.9 [8.7–14.4] cells/mm^2^; WHO II {III}, 13.6 [5.3–21.9] cells/mm^2^; WHO III, 18.1 [13.8–26.0] cells/mm^2^; WHO IV, 19.5 [14.7–24.9] cells/mm^2^; generalized linear model (quasipoisson), slope = 3.1 ± 0.8, *t* = 3.76, *P* = .002, *n* = 18 brain samples comprising 14 gliomas and 4 NGC controls) (Figure 2C). We concluded that, firstly, PpIX^+^ cells in HGGs were present at a higher density than in low-grade gliomas (LGGs) and, secondly, PpIX^+^ cells in LGGs tended to clump together to form cylindrical structures.

Molecular alterations in glioma cells are a better guide to clinical prognosis than the neuropathological WHO grade.^17^ Therefore, we investigated the relationship between PpIX fluorescence and the 2016 WHO molecular classification of gliomas.^18^ We found that differences in the density of PpIX^+^ cells across molecular subtypes, when not accounting for grade, were not statistically significant (median [IQR]: IDH1^mut^/1p19q^co-del^ 14.4 [8.7–21.1] cells/mm^2^; IDH1^mut^/1p19q^intact^ 18.0 [13.8–22.2] cells/mm^2^; IDH1^wt^ 19.0 [14.7–24.9] cells/mm^2^; one-way ANOVA, *F*_(2, 10)_ = 0.86, *P* = .45, *n* = 13 gliomas) (Figure 2D).

Collectively, our findings indicate that, firstly, PpIX^+^ cell density more closely reflects the WHO grade of the ex vivo samples rather than its molecular subtype and, secondly, PpIX^+^ cell density increases with malignant progression. This is consistent with the idea that metabolic changes in tumor cells become more pronounced as tumors become malignant.^10^

### PpIX Fluorescence Increases Sublinearly With Glioma Infiltration

**Figure 3.**
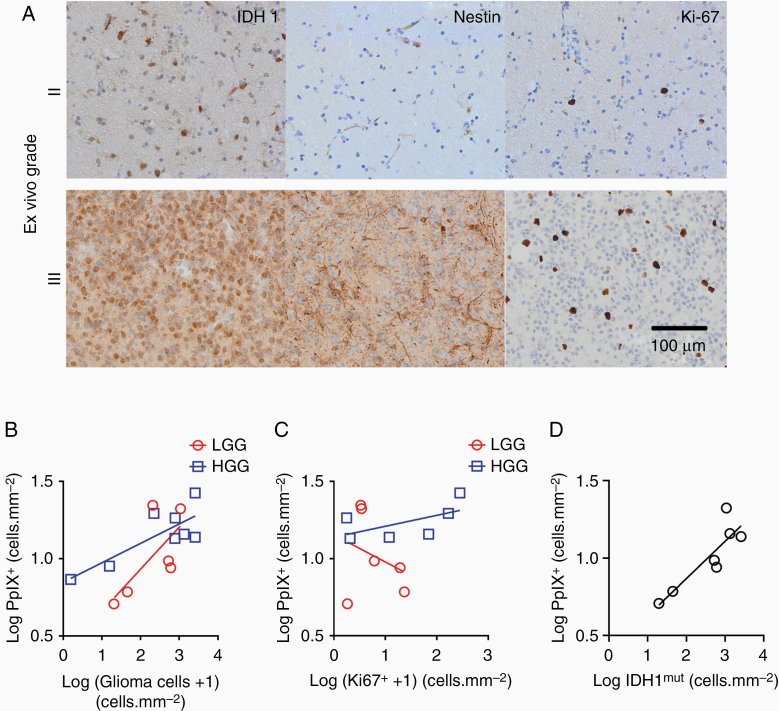
PpIX expression in low-grade and high-grade gliomas. (A) IDH1^mut^, Nestin, and Ki-67 stains of the edge of WHO II (upper panels) and WHO III gliomas (lower panels). (B) PpIX^+^ cell density increases with tumor infiltration in low-grade gliomas (LGG, red circles, 6 IDH1^mut^ gliomas) and in higher grade gliomas (HGG, blue squares, 6 IDH1^wt^ and 2 IDH1^mut^ gliomas). (C) No power relationship between PpIX^+^ cell density and Ki-67^+^ cell density in lower-grade gliomas (red line): LGG, 6 IDH1^mut^ gliomas; HGG, 4 IDH1^wt^ and 2 IDH1^mut^ gliomas. (D) Power relationship between numbers of PpIX^+^ cells and IDH^mut^ cells for all WHO glioma grades.

We next asked whether the density of PpIX^+^ cells was related to glioma cell infiltration or to the density of proliferating cells in LGGs and HGGs. Glioma cells were labeled for mutant IDH1 protein (IDH1^mut^) or for nestin.^19^ The ex vivo brain tissue samples were allocated to LGG and HGG groups based on the WHO grade of the diagnostic biopsies. We investigated whether the diagnostic grade of the glioma was related to cellular changes at its invasive edge (Figure 3A). We found a power relationship between PpIX^+^ cells and tumor infiltration for HGGs (Figure 3B; HGG slope = 0.13 ± 0.04, *R*^2^ = 0.65, *P* = .02, *n* = 8 WHO grade III or IV gliomas). However, there was no power relationship for LGGs (LGG slope = 0.27 ± 0.14, *R*^2^ = 0.48, *P* = .21, *n* = 6 WHO grade II or grade II {III} gliomas).

We next investigated whether the density of PpIX^+^ cells was related to the density of proliferating cells expressing Ki-67 in our ex vivo samples from the tumor edge, again using the diagnostic WHO groups. The density of PpIX^+^ cells in LGG and HGG did not increase with the density of proliferating cells (Figure 3C; LGG slope = −0.18 ± 0.29, *R*^2^ = 0.09, *P* = .57, *n* = 6 WHO grade II or II {III} gliomas; HGG slope = 0.07 ± 0.05, *R*^2^ = 0.35, *P* = .22, *n* = 6 WHO grade III or IV gliomas).

We revisited the relationship between PpIX fluorescence and glioma infiltration. WHO grading of a biopsy gives a snapshot of the cellular features of the glioma, but is a less accurate guide to prognosis than molecular profiling.^17^ The IDH1^mut^ molecular marker demarcates a subset of WHO grade II and grade III gliomas with a better prognosis.^17^ These WHO grade II and grade III gliomas are termed lower-grade.^17^ We reasoned that we could use the IDH1^mut^ molecular marker to reduce variability between gliomas and that this would give us a better way to assess whether the density of lower-grade glioma cells is related to PpIX^+^ cell density. We found a power relationship between the density of IDH1^mut^ cells and of PpIX^+^ cells in our ex vivo samples from lower-grade gliomas (Figure 3D; slope = 0.24 ± 0.06, *R*^2^ = 0.77, *P* = .009, *n* = 7 WHO grade II, II {III}, or III gliomas). The power coefficient of 0.24 indicated that the density of PpIX^+^ cells increases as approximately the fourth root of the density of lower-grade glioma cells. We concluded that PpIX^+^ cells increased with glioma cell number if the analysis was restricted to IDH1^mut^ lower-grade gliomas. However, the increase was markedly sublinear suggesting the glioma cell number is a weak determinant of PpIX fluorescence.

### Nestin^+^ Glioma Cells and CD34^+^ Endothelial Cells Contribute to PpIX “Hotspots” in IDH1^mut^ Diffuse Gliomas

**Figure 4.**
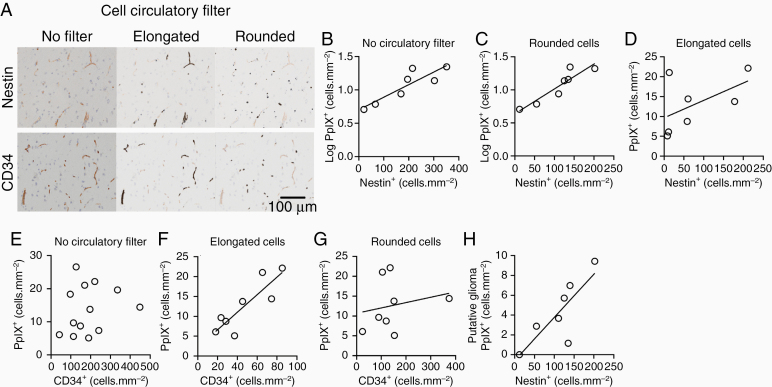
Cellular expression of PpIX at the edge of gliomas. (A) Shape (circulatory) filter divides image data for Nestin and CD34 stains (left panels) into elongated cells (middle panels) and rounded cells (right panels). (B) The number of PpIX^+^ cells grows exponentially as the number of nestin^+^ cells increases in WHO grade II tissue from the edge of WHO grade II and II{III} gliomas. (C) The exponential relationship between numbers of PpIX^+^ cells and rounded nestin^+^ cells at the edge of WHO grade II and II{III} gliomas. (D) No correlation between numbers of elongated nestin^+^ cells and PpIX^+^ cells in lower-grade gliomas. (E) No correlation between CD34^+^ cells and the density of PpIX^+^ cells in WHO grade II–IV gliomas when no shape filter is applied. (F) A Linear relationship between numbers of elongated CD34^+^ cells and PpIX^+^ cells in gliomas. (G) No correlation between numbers of PpIX^+^ cells and rounded CD34^+^ in gliomas. (H) The number of putative glioma cells that exhibit PpIX fluorescence increases with the number of nestin^+^ cells.

We had observed that PpIX fluorescence outlined vessel-like structures in control brain samples. Therefore, we investigated the contributions of glioma cells and endothelial cells to PpIX fluorescence. Our analysis had shown that PpIX fluorescence increases sublinearly as the number of IDH1^mut^ glioma cells increases. Therefore, we asked whether nestin^+^ cells in lower-grade IDH1^mut^ gliomas were linked to PpIX fluorescence. We found that the density of PpIX^+^ cells grew exponentially with the density of nestin^+^ cells (Figure 4A and B; slope = 0.002 ± 0.0004, *R*^2^ = 0.78, *P* = .008, *n* = 7 IDH1^mut^ gliomas). This suggests that nestin^+^ glioma cells contribute to PpIX fluorescence. However, endothelial cells may express nestin when replicating.^20^ Glioma cells tend to be rounded whereas endothelial cells tend to be elongated. Therefore, we applied a shape (circulatory) filter to separate the effects of elongated nestin^+^ cells from rounded nestin^+^ cells on PpIX fluorescence (Figure 4A and C). We found a strong relationship between rounded nestin^+^ cells and PpIX^+^ fluorescence at the edge of IDH1^mut^ lower-grade gliomas (Figure 4C; slope = 0.003 ± 0.001, *R*^2^ = 0.85, *P* = .003, *n* = 7 IDH1^mut^ gliomas), but no correlation with elongated nestin^+^ cells (Figure 4D; slope = 0.23 ± 0.03, *R*^2^ = 0.29, *P* = .22, *n* = 7 IDH1^mut^ gliomas). Taken together, our findings suggest that the density of PpIX^+^ cells in lower-grade gliomas grows exponentially as the density of nestin^+^ glioma cells increases.

We next studied the contribution of endothelial cells to PpIX hotspots by staining the tissue for endothelial cell marker, CD34 (Supplementary Figure 3C). We found no relationship when we compared the density of CD34^+^ cells with the density of PpIX^+^ cells in IDH1^mut^ gliomas (Figure 4E; *R*^2^ = 0.04, *P* = .51, *n* = 13 comprising 12 gliomas and 1 focal cortical dysplasia). However, CD34 is also expressed on reactive microglia and on a subset of glioma cells.^21,22^ Therefore, we applied the shape (circulatory) filter again to distinguish elongated endothelial cells from rounded microglia and glioma cells (Figure 4A). After applying the shape filter, we found a linear relationship between the density of putative CD34^+^ endothelial cells and the density of PpIX^+^ cells (Figure 4F; slope = 0.23 ± 0.05, *R*^2^ = 0.74, *P* = .006, *n* = 8 IDH1^mut^ gliomas). Endothelial cells that are entering into a proliferative state express nestin.^20^ However, application of the shape (circulatory) filter did not uncover a relationship between elongated nestin^+^ cells and PpIX^+^ cells in lower-grade gliomas (Figure 4D; slope = 0.02 ± 0.02, *R*^2^ = 0.07, *P* = .48, *n* = 9 IDH1^mut^ gliomas). Collectively, our data suggested that endothelial cells contributed to PpIX hotspots in lower-grade gliomas. However, the endothelial cells in PpIX hotspots were not proliferating in large numbers. Moreover, there was no correlation between the densities of rounded CD34^+^ cells and PpIX^+^ cells (Figure 4G; slope = slope = 0.01 ± 0.02, *R*^2^ = 0.06, *P* = .61, *n* = 8 IDH1^mut^ gliomas), suggesting the activated microglia were not major contributors to the PpIX signal.

Our data indicated that the PpIX^+^ cells in PpIX hotspots were a combination of nestin^+^ glioma cells and endothelial cells. Nestin is commonly used as a marker for glioma stem/progenitor cells.^23^ However, nestin is also expressed by other types of activated glial cells, such as reactive astrocytes and activated microglia.^24,25^ We estimated the PpIX^+^ cell density attributable to putative glioma cells by subtracting the average contribution of PpIX^+^ putative endothelial cells from the PpIX^+^ cell total (Supplementary Methods). In lower-grade IDH1^mut^ gliomas, we found that the number of PpIX^+^ putative glioma increased with the number of nestin^+^ cells (Figure 4H; slope = 0.04 ± 0.01, *R*^2^ = 0.65, *P* = .03, *n* = 7 IDH1^mut^ gliomas). We concluded that PpIX fluorescence is more common in nestin^+^ glioma cells.

### 5-ALA-Conjugated Quantum Dots Accumulate Around Blood Vessels and in Glioma Cells

**Figure 5.**
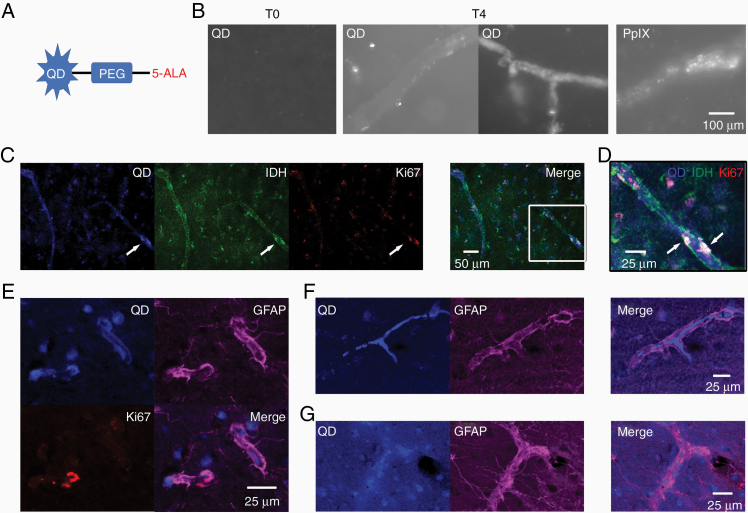
5-ALA-Quantum dot (5-ALA-QD) uptake in glioma cells and small blood vessels. (A) 5-aminolevulinic acid (5-ALA) was conjugated to quantum dot (QD) nanoparticles via a polyethylene glycol (PEG) linker. (B) 5-ALA-QD incubated with ex vivo brain slices. T0, before incubation. T4, after 4 h. Tubular structures were imaged with QD fluorescence or PpIX fluorescence ex vivo. (C) 5-ALA-QD, IDH1^mut^ and Ki-67 imaged in ex vivo slice. Arrows indicate fluorescence colocalization. The white box in the merged image enlarged in D. (D) Proliferating, lower-grade glioma cells (Ki-67^+^ and IDH1^mut^) that had taken up 5-ALA-QD adjacent to blood vessel-like structure. (E) 5-ALA-QD colocalize with GFAP^+^ cells. (F) Blood vessel-like structure outlined by GFAP staining shows the accumulation of 5-ALA-QD along the vessel wall. (G) Blood vessel-like structure outlined by GFAP staining that did not accumulate 5-ALA-QD.

Our data indicated that nestin^+^ glioma cells and endothelial cells contributed to PpIX fluorescence. We next investigated the spatial relationship of cells in PpIX hotspots. PpIX fluorescence photobleaches rapidly. Therefore, we conjugated 5-ALA to quantum dots (5-ALA-QD), which remain fluorescent after fixation, and incubated ex vivo human brain slices with 5-ALA-QD (Figure 5A and B).

Low levels of 5-ALA-QD were taken up by IDH1^mut^ glioma cells (Figure 5C). We observed IDH1^mut^ glioma cells that had accumulated 5-ALA-QD and were also proliferating next to blood vessels (Figure 5D). 5-ALA-QD were taken up by GFAP^+^ cell processes surrounding blood vessels (Figure 5E and F) and within the blood vessel wall (Figure 5E and G). These findings suggest that 5-ALA-QD are taken up by lower-grade glioma cells, astrocytic endfeet on vessel walls, and endothelial cells. We concluded that PpIX^+^ lower-grade glioma cells replicate in close proximity to blood vessels.

## Discussion

We used live human brain tissue to investigate the spatial origins of malignant progression in lower-grade gliomas. At the edge of lower-grade diffuse gliomas, we observed sparsely distributed clusters of 5-ALA-induced PpIX^+^ cells that we termed PpIX “hotspots.” These PpIX hotspots contained nestin^+^ glioma cells and endothelial cells. The cells in PpIX hotspots have characteristics, which suggest that PpIX hotspots may signify the earliest stages of malignant transformation.

High intracellular levels of PpIX indicate reprogramming of the heme biosynthesis pathway. This metabolic reprogramming is a feature of HGG cells and enables them to be visualized macroscopically during surgery.^12^ In contrast, only a minority of LGGs exhibit macroscopic PpIX fluorescence and, when present, are thought to indicate that malignant progression has occurred.^16,26,27^ This is consistent with the idea that metabolic reprogramming becomes more pronounced with malignant progression.^10^ Our data suggest that microscopic PpIX hotspots are present in lower-grade glioma tissue in the absence of macroscopic PpIX fluorescence and without marked increases in proliferation.

We found that the densities of PpIX^+^ cells increased exponentially with the density of nestin^+^ cells in lower-grade gliomas. In the healthy central nervous system, nestin is expressed by neural progenitor/stem cells, but not by differentiated neurons and glial cells.^28,29^ Nestin has been used as a marker for glioma stem cells in glioblastomas.^23^ More work is needed to quantify the proportion of IDH1 cells that are PpIX^+^ and nestin^+^. However, our data suggest that a subset of PpIX^+^ lower-grade glioma cells not only exhibit metabolic reprogramming, but are also in a less differentiated cellular state.

Transcriptomic studies indicate that diffuse lower-grade gliomas originate from neural progenitor-like cells.^30,31^ These neural progenitor-like cells replicate, unlike the vast majority of diffuse lower-grade glioma cells.^30,31^ Our data indicate that the glioma cells in PpIX hotspots can divide. Therefore, our findings combined with the transcriptomic studies^30,31^ suggest that PpIX hotspots include neural progenitor-like cells.

We found that endothelial cells contributed to PpIX hotspots in ex vivo lower-grade glioma tissue. Notably, our neuropathological and molecular data indicate that the endothelial cells in PpIX hotspots were not proliferating. Our results are consistent with experiments on endothelial cell cultures that have shown that quiescent endothelial cells accumulate PpIX, although to a lesser extent than proliferating endothelial cells.^32^

The PpIX hotspots frequently had a tubular or branched structure resembling blood vessels. We found no evidence of microvessel proliferation in WHO grade II glioma tissue. This suggests that the glioma cells in PpIX hotspots were growing along established blood vessels, which is termed vessel co-option.^33,34^ Molecules released by endothelial cells of small blood vessels create perivascular niches, which attract and sustain glioma cells.^35–37^ Niches have been subdivided on the basis of the signaling mechanisms operating within the niche.^38^ Our data suggest that the PpIX hotspots in lower-grade gliomas represent an invasive niche involving nestin^+^ glioma cells, which may proliferate around small blood vessels.

The glioma cells in the PpIX hotspots exhibit several hallmarks of cancer, such as less differentiated cellular state, ability to divide, and metabolic reprogramming, which indicate high-grade potential and suggest that PpIX hotspots may be seedbeds for malignant progression. PpIX hotspots were sparse, but widely distributed, suggesting that malignant progression can start at many locations within lower-grade gliomas. Each PpIX hotspot provides a microdomain, which could fuel malignant transformation. Understanding the spatial origins of malignant progression at a cellular level will provide a basis for new treatments to prevent it and to reduce the risk of glioma recurrence after resective surgery.

## Supplementary Material

vdab026_suppl_Supplementary_Material

## Notes

**Authorship Statement.** A.J.K. and G.T.F. designed the study. J.P.L., F.V., R.B., and K.A. collected samples or data. A.J.K. did the experimental work. A.J.K. and G.T.F. analyzed the data. I.B. made a neuropathological diagnosis from fixed samples. A.J.K. and G.T.F. wrote the manuscript with input from J.P.L., I.B., F.V., R.B., and K.A.
