# Supplementary material for: Multicellular “hotspots” harbor high-grade potential in lower-grade gliomas
Source: Neurooncol Adv. 2021 Feb 8;3(1):vdab026. doi: 10.1093/noajnl/vdab026 (PMC8082133; doi:10.1093/noajnl/vdab026)
Supplement: vdab026_suppl_Supplementary_Material [file vdab026_suppl_supplementary_material.docx]

**SUPPLEMENTARY INFORMATION**

**Supplementary Methods**

*Immunofluorescence staining of fixed human brain tissue*

After imaging, brain slices were fixed in 4 % PFA for 20-30 minutes and washed three times in 1 mM PBS before re-sectioning into 50 µm thick sections using a VT1000S vibratome (Leica Biosystems). These sections were then incubated in 0.3 % Triton X-100 and 3 % Normal Goat Serum for 2 hours. The primary antibody (Supplementary Table 1) was incubated in 0.3 % Triton X-100 and 2 % BSA at ^+^4 ^o^C for 24 hours. After incubation with the primary antibody the sections were washed 3 times in 1 mM PBS. If secondary antibodies were required, the sections were incubated in the secondary antibodies with 0.3 % Triton X-100 and 3 % BSA for 2 hours (Supplementary Table 1). Following 3 more washes in 1 mM PBS, the slices were mounted using VectaShield Prolong Diamond antifade (ThermoFisher: P36965).

*Supervised cell counts*

Supervised cell counting was done using a custom written script in Fiji.

i) PpIX^+^ cells

The mean background was estimated from a 50-pixel radius area and was subtracted from the entire fluorescence image (8 bit). Image contrast was then enhanced in FIJI such that 0.1% of the pixels become saturated. A maximum entropy threshold in FIJI Particle Analysis was applied to the image to extract PpIX^+^ particles (Supplementary Fig. 1Ai, Aii). A high intensity threshold was chosen to select PpIX positive particles near the surface of thick *ex vivo* human brain slices (Supplementary Fig. 1). This more closely mirrors the data obtained from thin neuropathology sections. A PpIX^+^ particle was counted as a cell if the particle area was between 40 µm^2^ and 500 µm^2^ (Supplementary Fig. 1Aiii, B).

ii) Nestin and CD34

Neuropathological slides were scanned on an Olympus VS120 Slide scanner. A threshold for pixel intensity of 110 was set for the images (8 bit) to extract nestin^+^ particles (Supplementary Fig. 2A, B) or CD34^+^ particles. Nestin^+^ particles were counted as cells if the particle area was between 20 µm^2^ and 500 µm^2^, which selects for somata and their large processes (Supplementary Fig. 2C, D). A circularity filter was applied to the extracted particles, where circularity = 4π(area/perimeter^2^). Particles with a circularity of 0 - 0.3 were referred to as elongated and particles with a circularity of 0.3 – 1 were deemed round.

*5-ALA conjugated quantum dots*

We conjugated 5-ALA to fluorescence quantum dots nanocrystals via a polyethylene glycol (PEG) linker to enable us to image cells that had taken up 5-ALA after fixation. Qdot® 525 ITK™ amino (PEG) quantum dots (ThermoFisher: Q21541MP) were crosslinked with the carboxylic acid group in 5-ALA. Before conjugation, 1 nM Qdots were suspended in 1 mM phosphate buffered saline and underwent centrifugal 100 kDa ultrafiltration. Bis[sulfosuccinimidyl] substrate (BS3) (A39266: ThermoFisher) was then added to the filtrated Qdots in a ratio of 10 µM / 1 mM BS3 for 30 minutes. The Qdots were purified using a NAP-5 column (Fisher Scientific) that was pre-equilibrated using 1 mM PBS. A 40-fold excess of 5-ALA was added to the purified Qdots and left to react on a rotator at room temperature for two hours. The reaction was then quenched by applying 50 mM glycine for 15 minutes. The conjugate then underwent 100 kDa ultrafiltration, before passing through a 0.8 / 0.2 µm filter.

**Supplementary Table 1: Antibody list**

| **Primary antibody** | **Concentration** | **Secondary antibody** | **Concentration** |
| --- | --- | --- | --- |
| Chicken anti-GFAP (Abcam; ab4674) | 1:1000 | Goat anti-chicken, 647 (ab150171) | 1:1000 |
| Rat anti IDH 1/2 mutated  (Bio serv: MBL-D332-3) | 1:500 | Goat anti-rat, 647  (Abcam; ab150159) | 1:1000 |
| Alexa Fluor 647 Ki-67 anti-human  (Bio legends; 350510) | 2.5 µg per ml | N/A | N/A |
| Alexa Fluor 488 Ki-67 anti-human  (Bio legends; 350532) | 2.5 µg per ml | N/A | N/A |

Abbreviations: GFAP, Glial Fibrillary acid protein; IDH 1/2, isocitrate dehydrogenase 1/2.

**Supplementary Table 2: Data used in Figure panels**

| **ID** | **Tumor Information** | | |  |
| --- | --- | --- | --- | --- |
|  | **Tumor / Diagnostic grade** | **Ex vivo grade** | **Molecular Markers** | **Figure Panels** |
| **1** | Oligo  III | II | IDH1^mut^, 1p/19q co-del,  ATRX retained | 2C, 2D, 3B – D, 4B - H |
| **2** | Oligo  II | II | IDH1^mut^, 1p/19q co-del,  ATRX retained, TERTp^mut^, | 2C, 2D, 3B |
| **3** | Oligo  III | III | IDH1^mut^, 1p/19q co-del, ATRX retained | 2C, 2D, 3B – D, 4B - H |
| **4** | Oligo  III | III | IDH1^mut^, 1p/19q co-del, ATRX retained | 2C, 2D |
| **5** | Astro  III | II | IDH2^mut^ (R132C),  ATRX lost, TERTp^wt^ | 2C, 2D, 3B – D, 4B - H |
| **6** | Oligo  III | II | IDH1^mut^, 1p/19q co-del, ATRX retained, TERTp^mut^ | 2C, 2D, 3B – D, 4B - H |
| **7** | Oligo  II | II | IDH1^mut^, 1p/19q co-del, ATRX retained, TERTp^mut^ | 2C, 2D, 3B – D,  4E - G |
| **8** | Oligo  II | II | IDH1^mut^, 1p/19q co-del, ATRX retained | 2C, 2D, 3B – D, 4B - H |
| **9** | Astro  III | III | IDH1^mut^,  ATRX lost, TERTp^wt^ | 2C, 2D, 3B – D, 4B - H |
| **10** | Oligo  III | II | IDH1^mut^, 1p/19q co-del, ATRX retained | 2C, 2D, 3B – D, 4B – H,  5C – E, 5G |
| **11** | GBM  IV | IV | IDH1^wt^,  ATRX retained | 2C, 2D  3B, 3C, 4E |
| **12** | GBM  IV | IV | IDH1^wt^,  ATRX retained, TERTp^mut^ | 2C, 2D  3B, 3C |
| **13** | GBM  IV | No glioma cells | IDH1^wt^,  ATRX retained, TERTp^mut^, | 2C, 2D  3B |
| **14** | GBM  IV | No glioma cells | IDH1^wt^, TERTp^mut^ | 2C, 2D  3B, 4E |
| **15** | GBM  IV | IV | IDH1^wt^,  ATRX retained, TERTp^mut^, | 2C, 2D  3B, 3C, 4E |
| **16** | GBM  IV | IV | IDH1^wt^,  ATRX retained, TERTp^mut^, | 2C, 2D  3B, 3C, 4E |
| **17** | Focal Cortical Dysplasia | No glioma cells | CD34 Negative | 2C, 2D  4E |
| **18** | Astro  III | III | IDH1^mut^,  ATRX lost | 5B |
| **19** | Metastatic  adenocarcinoma | No glioma cells | Not applicable | 2C, 2D |
| **20** | Astro  III | III | IDH1^wt^,  ATRX retained, TERTp^wt^ | 5B, 5F |

**Supplementary Figure 1**

**
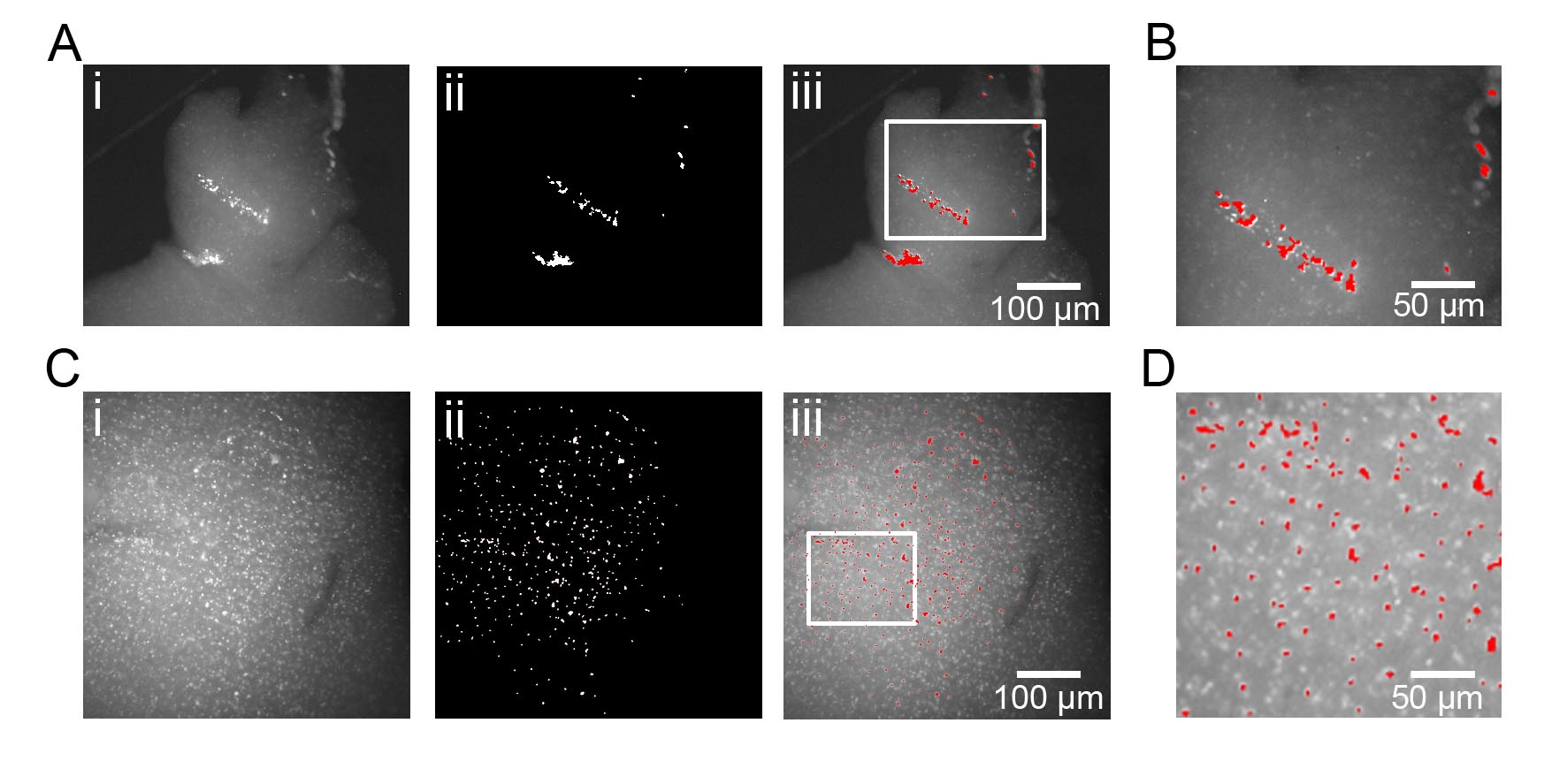
**

**Supervised counting of PpIX^+^ cells in *ex vivo* human brain samples**

**A)** Low-grade glioma. i) Fluorescence image of brain slice, ii) fluorescent cells extracted in FIJI, iii) merged images with fluorescent cells coloured red. **B)** Enlarged image of region demarcated by white box in Aiii. **C)** Glioblastoma. Left; Raw image middle; overlay processed in FIJI right; merge of both images, red masks are particles counted in FIJI. **D)** Enlarged image of region demarcated by white box in Ciii.

**Supplementary Figure 2**

**
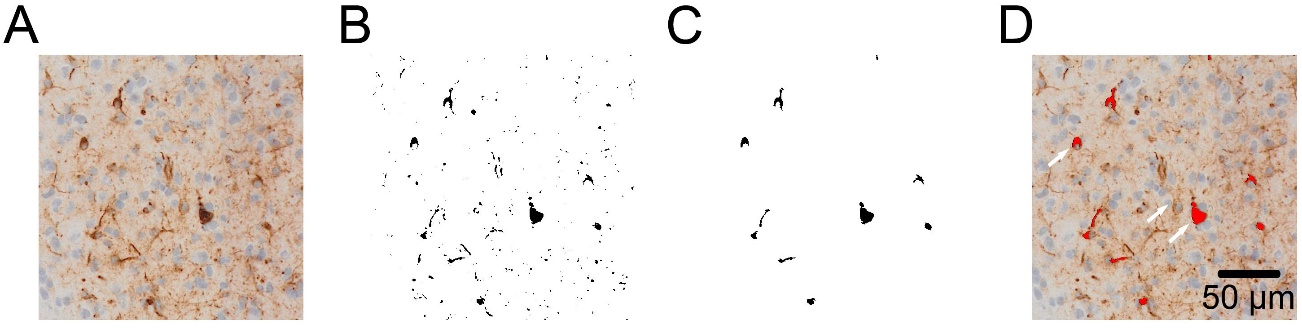
**

**Supervised counting of Nestin^+^ cells**

**A)** Anaplastic oligodendroglioma (WHO grade III) stained for nestin. **B)** Threshold analysis reveals nestin^+^ particles. **C)** Size filter separates nestin^+^ cell bodies and largest processes from smaller irregular particles. **D)** Nestin^+^ particles extracted by analysis (red) overlaid on source image in panel A. White arrows indicate Nestin^+^ cell body and false negative cell with nestin staining below threshold. Scale bar, 25 µm all images.

**Supplementary Figure 3**

**
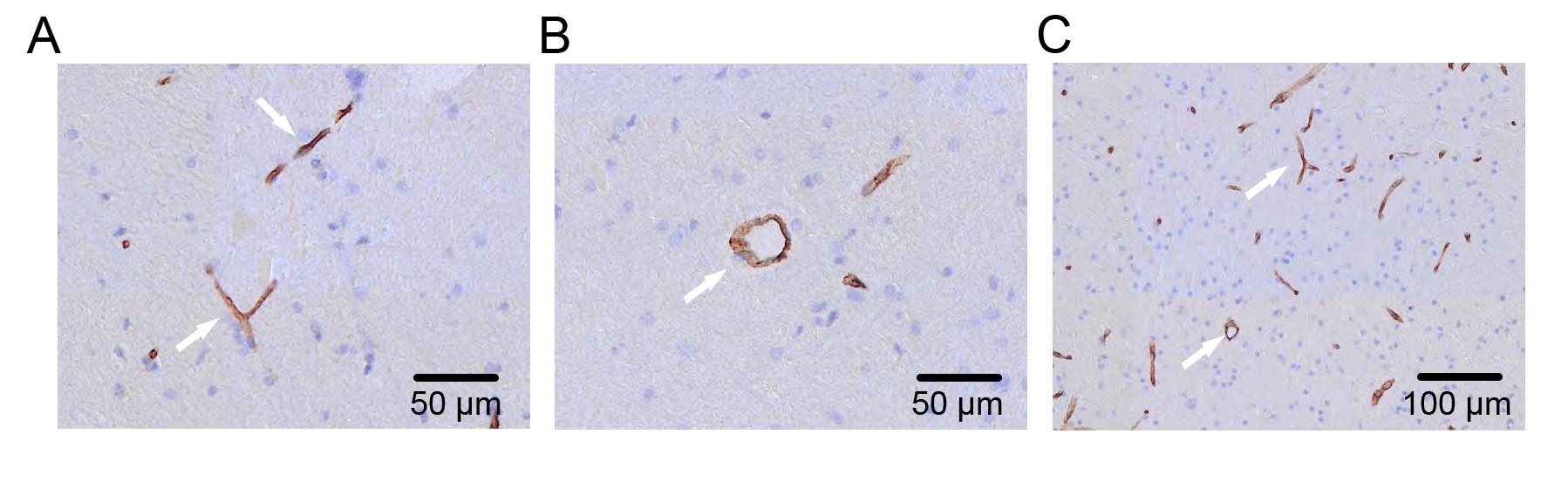
**

**Intracerebral blood vessels stained with CD34 in thin sections cut through *ex vivo* human brain samples**

CD 34 staining of thin sections highlights the linear and branching patterns of capillaries (**A**) and venules (**B**) in an *ex vivo* sample characterised as “no glioma cells” (NGC, < 10 glioma cells.mm^-2^) donated by a patient with a glioblastoma. **C**) capillaries and venule (lower arrow) in an *ex vivo* sample from patient with a WHO Grade II Oligodendroglioma
